# Long-term survival following neoadjuvant chemotherapy and concomitant radiochemotherapy in locally advanced cervical cancer: results of the Oncology Institute "Prof. Dr. Ion Chiricuta" experience

**Published:** 2018

**Authors:** Andreea Marita, Claudia Ordeanu, Alin Rancea, Todor Nicolae, Viorica-Magdalena Nagy

**Affiliations:** * Oncology Institute “Prof. Dr. Ion Chiricuta” Cluj- Napoca; ** Iuliu Hatieganu University of Medicine and Pharmacy Cluj Napoca

**Keywords:** Cervical carcinoma, Concurrent chemoradiotherapy, Neoadjuvant chemotherapy, Radical hysterectomy, Survival

## Abstract

**Objective:**to analyze the efficiency of (NACT) followed by concurrent radiochemotherapy (RCT) in patients with locally advanced cervical cancer, 5-year overall, specific and disease-free survival and the prognostic factors correlated with the response and survival.

**Materials and methods:**207 patients with cervical carcinoma stages IIB-IIIB, who received 2-4 cycles of neoadjuvant chemotherapy followed by concurrent chemoradiation were retrospectively analyzed for an objective response (OR), overall survival (OS), and disease-specific survival (DSS) rate. All patients received platinum-based NACT followed by concurrent RCT to a total dose (TD) of 46 Gy/pelvis when patients were evaluated for surgery. Patients with favorable parametrial response optionally underwent surgery. The rest of the patients continued radiochemotherapy exclusively.

**Results:**The baseline characteristics were: median age at diagnosis - 52 years; 82% squamous and 12% adenocarcinoma histologies; 67 patients (32.4%) with FIGO stage IIB, 87 (42%) with stage IIIA and 53 (25.6%) with stage IIIB. The OR rate was 56.5% post-NACT and the complete response (CR) after exclusive RCT was 19.7% while pathological complete response (pCR) in patients that underwent surgery was 61.2%. The median follow-up was 58.3 months. Overall and disease-specific survivals at 5 years were 78% and 84%, respectively. The OS for stages IIB and IIIA was 84%, and 61% for stage IIIB while the DSS rates were 90% for stage IIB, 86% for stage IIIA and 72% for stage IIIB. The disease-free intervals (DFS) rates were 88%, 76% and 69% for stages IIB, IIIA and IIIB, respectively.

**Conclusions:**Neoadjuvant chemotherapy followed by concurrent chemoradiation produces higher response rates and improvements in disease-specific survival and disease-free survival rates compared to RCT.

## Introduction

Cervical cancer is ranked 4th in the world regarding malignant tumors in women, accounting for 12% of all cancers in women. 85% of cases occur in developing countries, where they are the leading cause of death from cancer among women. The global annual incidence of cervical cancer in 2012 was 528.000. In 2012, 266.000 deaths from cervical cancer worldwide were estimated, accounting for 7.5% of all female cancer deaths [**1**]. In Romania, cervical cancer accounts for 15% of all malignant tumors, being the first cause of female death in women in female genital cancers and third after breast cancer and colorectal cancer in other types of cancers. Unfortunately, our country ranks first in Europe regarding cervical cancer mortality [**2**].

Concomitant radiotherapy and chemotherapy (CRCT) are considered the standard combination of treatment for locally advanced cervical cancer. With 5-year overall survival ranging from 60% to 65% for stage IIB and from 25% to 50% for stage III, new therapeutic strategies are investigated in order to improve these results [**3**]. Neoadjuvant chemotherapy (NACT) prior to surgery or radiotherapy has been investigated as a new therapeutic strategy for a voluminous or locally advanced disease. The reasons for using neoadjuvant chemotherapy (NACT) are several. Reducing the size of the tumor can later facilitate local therapy, either radiotherapy or surgery. This reduction can convert inoperable tumors to resectable ones. It has also been suggested that NACT increases the radiosensitivity and decreases the fraction of hypoxic cells. Moreover, NACT treats the micrometastatic disease, preventing a significant proportion of relapses. Finally, the NACT response was identified as an important prognostic factor in several studies [**4**][**5**].

The objective of this retrospective study was to analyze the efficacy of neoadjuvant chemotherapy followed by concurrent chemoradiation in patients with locally advanced cervical cancer, 5-year overall, specific survival and the prognostic factors correlated with the response and survival.

## Material and methods

The patients included in this study had histologically confirmed stage IIB-III cervical cancer (FIGO staging modified by MD Anderson Cancer Center) [**6**] treated at the Institute of Oncology “Prof. Dr. Ion Chiricuta” Cluj- Napoca between November 2010 – December 2013. Clinical staging was performed using pelvic examination by an experienced gynecologic oncologist and a radiation oncologist. Before treatment, all patients underwent several investigations as part of the initial workup: hematology and biochemistry profiles, chest X-ray, abdominal and pelvic computed tomography. All patients signed the informed consent before treatment. After diagnosis, patients recieved two to four cycles of NACT, two regimens of Paclitaxel 175mg/m2 and Carboplatin AUC 5 (PC) or Topotecan 0.75mg/m2 and Cisplatin 50mg/m2 (TC) administered every three weeks. Three weeks after the completion of the last NACT cycle, patients started RCT. The radiosensitizer chemotherapeutic agents administered concurrently with external beam radiation therapy (EBRT) were two regimens of Cisplatin (20mg/m2 for 5 days every three weeks – for 92.8% (192/207) of the patients) or 40mg/m2 weekly – 4.8%(10/207)] or Carboplatin AUC 2 – 2.4% (5/207).

External beam radiotherapy (EBRT) to the abdominopelvic region was delivered using 15-MV x-rays. Radiation was given in daily fractions of 2 Gy for 5 days/week using the 4-field technique (conventional (2D) or conformal (3D) radiotherapy), to a total dose of 46 Gy/23 fractions. In patients with exclusive RCT after 46 Gy, the EBRT was continued with a small 4-field pelvic boost at the total dose of 60 Gy. A cervical boost was given using intracavitary brachytherapy at a total dose of 10 Gy preoperatively or 20 Gy for patients with exclusive RCT or using the x-ray arch technique at a total dose of 10 Gy preoperatively or 14 Gy for patients with exclusive RCT. At 46 Gy on the pelvis + 10 Gy per cervical boost, patients were clinically evaluated according to the local response. The patients with a favorable parametrial response (reduction of the lateral parametrial invasion) optionally underwent completion surgery or continued concomitant RCT. The possibility to perform surgery was evaluated following a gynecological examination. All the results were multidisciplinary discussed. Surgery (radical abdominal hysterectomy with bilateral pelvic lymphadenectomy) was performed 6 to 8 weeks after the end of preoperative RCT. Patients with the decision of exclusive RCT immediately (after the decision) continued radiotherapy (RT) until the total dose of 60 Gy/30 fractions on the pelvis and 20 Gy per cervical boost with concomitant chemotherapy (CT).

Tumor response was evaluated according to the RECIST v1.1 criteria [**7**] defined as complete response-CR, partial response-PR, stable disease-SD and progressive disease-PD by a pelvic examination three weeks after the administration of the last NACT cycle, before RCT and at the end of RCT. For the operated patients, the tumor response was confirmed by the pathological outcome. Pathological CR was defined as the absence of tumor cells in the cervix, parametrium, vaginal cuff and pelvic lymph nodes. Patients were followed up periodically. Follow-up was scheduled at 3 months for the first year after treatment, every 6 months for the next 2 years, then once a year thereafter. The follow-up was performed at a multidisciplinary consultation (by both a gynecologic oncologist and a radiation oncologist). Local and distant control was evaluated at every visit by gynecologic and general clinical examination. Medical imaging was performed if recommended.

Local failure was defined as the occurrence of tumor relapse in the uterus, parametrium, or vagina; any other failure in the pelvis was considered as regional while any failure elsewhere was considered as distant.

Patient survival was measured from the start of treatment to the date of the last follow-up examination or death. The Kaplan-Meier method was used to estimate the survival rate. The statistical significance of the survival curves was calculated by the log-rank test. Comparison of categorical variables was performed using the χ2 test. P < 0.05 was considered statistically significant.

## Results

**Patient characteristics**

A total of 207 patients were included in this retrospective study. The median age was 52 years (ranging from 22 to 73 years). The median tumor diameter was 4 cm (ranging from 1 to 8 cm). There were 67 patients (32.4%) with stage IIB disease, 87 (42%) with stage IIIA disease, and 53 (25.6%) with stage IIIB. A majority of patients (182/207, 87.9%) had squamous cell carcinoma. Patient characteristics are shown in **Table 1**.

**Table 1:** Patient characteristics

	Overall	NACT+RCT+Surgery	NACT+RCT
**No. (%) patients**	207	85	122
**Median age (range), y**	52 (22-73)	48 (22-67)	54 (28-73)
**FIGO stage**
IIB	67 (32.4)	55 (64.7)	12 (9.8)
IIIA	87 (42)	27 (31.8)	60 (49.2)
IIIB	53 (25.6)	3 (3.5)	50 (41)
**Median tumor size (range), cm**	4 (1-8)	4 (1-8)	4.5 (1-7)
**Histology, n (%)**
Squamous cell carcinoma	182 (87.9)	75 (88.2)	107 (87.7)
Adenocarcinoma	25 (12.1)	10 (11.8)	15 (12.3)
**NACT type- n (%)**
Paclitaxel + Carboplatin	177 (85.5)	72 (84.7)	105 (86)
Topotecan + Cisplatin	30 (14.5)	13 (15.3)	17 (14)
**NACT No.of cycles n (%)**
1 cycle	10 (4.8)	1 (1.2)	9 (7.4)
2 cycles	71 (34.3)	28 (33)	43 (35.2)
3 cycles	95 (45.9)	44 (51.8)	51 (41.8)
4 cycles	31 (15)	12 (14)	19 (15.6)
**Objective response to NACT n (%)**
CR	4 (1.9)	2 (2.3)	2 (1.6)
PR	113 (54.6)	43 (50.6)	70 (57.4)
SD	90 (43.5)	40 (47.1)	50 (41)

**Objective response to NACT and CRCT**

85.5% (177 patients) out of the 207 patients underwent NACT with PC (Paclitaxel + Carboplatin) and 14.5% (30 patients) underwent NACT with TC (Topotecan + Cisplatin). 117 patients (56.5%) achieved an objective response (OR= CR+PR) after the administration of NACT. Regarding the administered NACT regimen, we observed a 59.9% OR (106/117) for PC versus 36.7% (11/30) for TC, with statistical significance (p=0.02) (**Table 3**). When evaluating the response to NACT in comparison with the number of administered NACT cycles, the OR was 53.4% with the administration of two cycles, 68.4% and 64.5% with the administration of three and four PC cycles, respectively.

Regarding the TC regimen, the OR was 30.8% after the administration of two cycles versus 37.5% after the administration of three cycles (**Table 2**).

**Table 2:** Response to neoadjuvant chemotherapy (regimen and number of cycles administered)

		Therapeutic response after neoadjuvant chemotherapy n (%)		
Neoadjuvant chemotherapy regimen		OR (CR+PR)	SD	Total
**Paclitaxel & Carboplatin**				
	1 cycle	1 (11.1)	8 (88.9)	9
	2 cycles	31 (53.4)	27 (46.6)	58
	3 cycles	54 (68.4)	25 (31.6)	79
	4 cycles	20 (64.5)	11 (35.5)	31
**Topotecan & Cisplatin**				
	1 cycle	1 (100)		1
	2 cycles	4 (30.8)	9 (69.2)	13
	3 cycles	6 (37.5)	10 (62.5)	16
	4 cycles			
	**Total**			207

**Table 3:** Clinical parameters associated with neoadjuvant chemotherapy response

Variable		No. of patients	Responders n (%)	Non responders n (%)	p value	
			CR+PR	ST+PD
**FIGO stage**					
	IIB	67	37 (55.2)	30 (44.8)	0.70
	IIIA	87	52 (59.8)	35 (40.2)	
	IIIB	53	28 (47.2)	25 (47.2)	
**Tumor size (baseline)**	
	≤ 4 cm	107	67 (62.6)	40 (37.4)	0.07
	> 4 cm	100	50 (50)	50 (50)	
**Age**	
	≤ 44 years	54	24 (44.4)	30 (55.6)	0.04
	> 44 years	153	93 (60.8)	60 (39.2)	
**Hb level (initial)**	
	≤ 12 mg/dl	39	16 (41)	23 (59)	0.03
	> 12 mg/dl	168	101 (60.1)	67 (39.9)	
**Neoadjuvant chemotherapy regimen**	
	Paclitaxel + Carboplatin	177	106 (59.9)	71 (40.1)	0.02
	Topotecan + Cisplatin	30	11 (36.7)	19 (63.6)	
**NACT No.of cycles**	
	1-2 cycles	81	37 (45.7)	44 (54.3)	0.01
	3-4 cycles	126	80 (63.5)	46 (36.5)	
**Histological type**	
	Squamous cell carcinoma	182	107 (58.8)	75 (41.2)	0.08
	Adenocarcinoma	25	10 (40)	15 (60)	
**Cervical boost**	
	BT	62	39 (62.9)	23 (37.1)	0.53
	Arch technique	20	11 (55)	9 (45)	
		3	2 (66.7)	1 (33.3)	

The correlation between the clinical parameters and the NACT response is summarized in **Table 3**. Stage IIIA disease had a more favorable response (59.8%) to NACT when compared to the response in stages IIB (55.2%) and IIIB (52.8%), respectively, the difference is not statistically significant (p=0.70). However, there were no significant differences in tumor size and histological type between NACT responders and non-responders. The univariate analysis showed a statistically significant difference in age, initial Hb level, type of chemotherapy (PC was superior to TC) and the number of cycles (3-4 cycles were more appropriate than 1-2 cycles) between NACT responders and non-responders.

All patients were evaluated after concurrent radiochemotherapy at a total dose of 46 Gy. A CR after CRCT was obtained in 25 (12.1%) patients. FIGO stage and tumor size were significantly correlated with the clinical outcome. After concurrent radio-chemotherapy, stage IIB disease had a more favorable response (22.4%), when compared with the response in stages IIIA (10.4%) and IIIB (1.2%), respectively (p<0.01). Patients with baseline tumor volume ≤ 4 cm had statistically significant higher CR rate compared to patients with baseline tumor volume > 4 cm (p<0.01). The correlation between the clinical parameters and CRCT response is summarized in **Table 4**.

**Table 4:** Clinical parameters associated with concurrent radiochemotherapy response

Variable		No. of patients	Responders n (%)	Non responders n (%)	p value	
			CR+PR	ST+PD
**FIGO stage**					
	IIB	67	15 (22.4)	52 (77.6)	<0.01
	IIIA	87	9 (10.4)	78 (89.7)	
	IIIB	53	1 (1.2)	52 (98.1)	
**Tumor size (baseline)**					
	≤ 4 cm	107	18 (16.8)	89 (83.2)	0.03
	> 4 cm	100	7 (7)	93 (93)	
**Age**					
	≤ 44 years	54	3 (5.6)	51 (94.4)	0.09
	> 44 years	153	22 (14.4)	131 (85.6)	
**Hb level (initial)**					
	≤ 12 mg/dl	39	3 (7.7)	36 (92.3)	0.51
	> 12 mg/dl	168	22 (13.1)	146 (86.9)	
**Neoadjuvant chemotherapy regimen**					
	Paclitaxel + Carboplatin	177	24 (13.6)	153 (86.4)	0.20
	Topotecan + Cisplatin	30	1 (3.3)	29 (96.7)	
**NACT No. of cycles**					
	1-2 cycles	81	8 (9.9)	73 (90.1)	0.44
	3-4 cycles	126	17 (13.5)	109 (86.5)	
**Histological type**					
	Squamous cell carcinoma	182	22 (12.1)	160 (87.9)	0.75
	Adenocarcinoma	25	3 (12)	22 (88)	
**Cervical boost**					
	BT	89	19 (21.3)	70 (78.7)	0.44
	Arch technique	33	5 (15.2)	28 (84.8)	

Radical surgeries would be performed optionally on the patients whose diseases were judged to be radically resectable with free margins. 85 (41%) patients underwent radical hysterectomy and pelvic lymph node dissection, and 122 continued exclusive RCT. Pathological evaluation revealed pCR in 52 (61.2%) of the operated patients and 33 (38.8%) were with positive pathologic findings. A median number of 19 lymph nodes (ranging from 6 to 46) were resected. Pathologic positive outcome was as follows: 27% (23/85) had residual tumor cells only at the uterine cervix, 2.4% (2/85) had only positive lymph nodes, and 9% (8/85) had residual tumor cells at the level of the uterine cervix, parametrial tissue, vaginal margins, endometrium, ovary and/or lymph nodes. Univariate statistical analysis indicates a more frequent pCR in patients with stage IIB, tumor size <4.5 cm, age > 44 years but without statistical significance. In stage IIB a 63.6% (35/55) pCR was achieved. 30 stage III patients underwent surgery and 17 patients achieved pCR, of which 14 were identified with stage IIIA disease.

122 (59%) patients received exclusive RCT. Evaluation at the end of RCT showed a CR in 19.7% of cases (24 patients) while 98 patients (80.3%) were non-complete responders (NCR). After concurrent radio-chemotherapy, stage IIB disease had a favorable response (25%), when compared with the response in stages IIIA (21.7%) and IIIB (16%), respectively. CR was revealed in 22.9% of patients in the Paclitaxel and Carboplatin group and none in the Topotecan and Cisplatin group. A higher rate of CR has been accomplished in the case of patients with tumors ≤ 4.5 cm (p=0.01). In a univariate analysis, we evaluated the correlation of the main factors studied with the outcome of the concomitant RCT. There were no significant differences in the FIGO stage, age, initial Hb level, chemotherapy type, number of cycles, and histological type between complete responders and NCR, respectively.

**Survival**

**Fig. 1:**
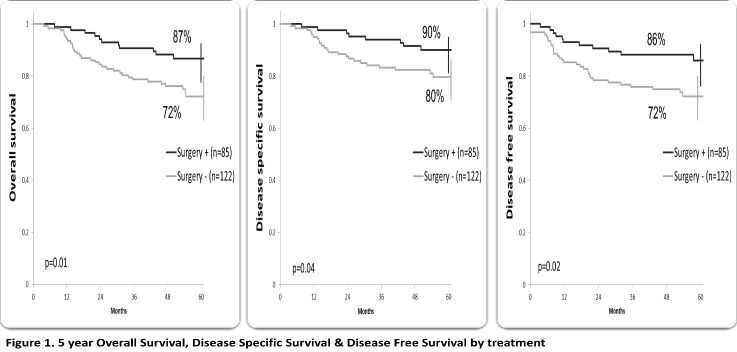
5-year Overall Survival, Disease-Specific Survival & Disease-Free Survival by treatment

**Fig. 2:**
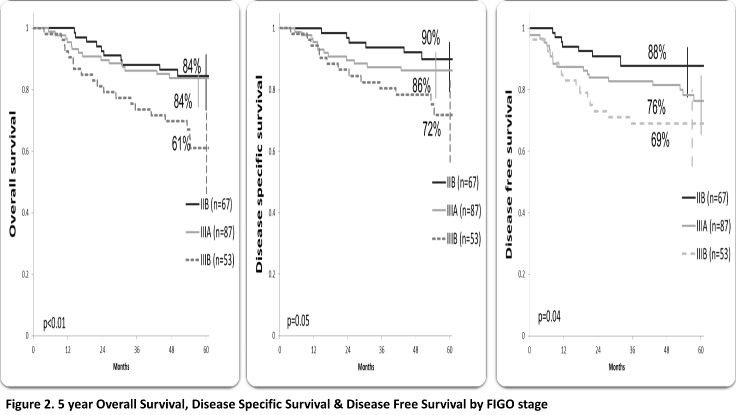
5-year Overall Survival, Disease-Specific Survival & Disease-Free Survival by FIGO stage

**Fig. 3:**
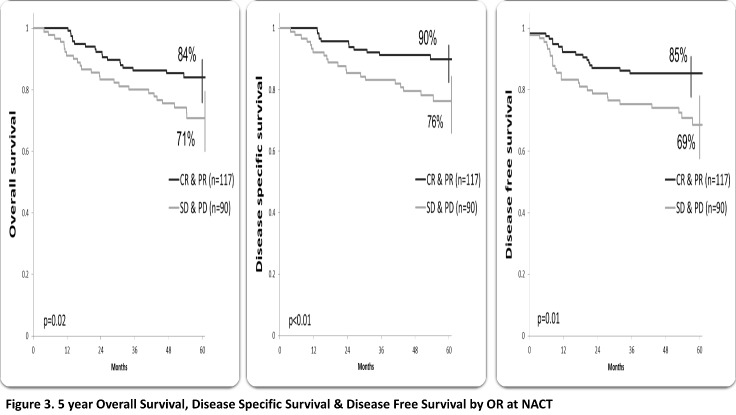
5-year Overall Survival, Disease-Specific Survival & Disease-Free Survival by OR at NACT (CR=complete response, NACT=neoadjuvant chemotherapy, OR=objective response, PR=partial response, PD=progressive disease, SD=stable disease)

**Fig. 4:**
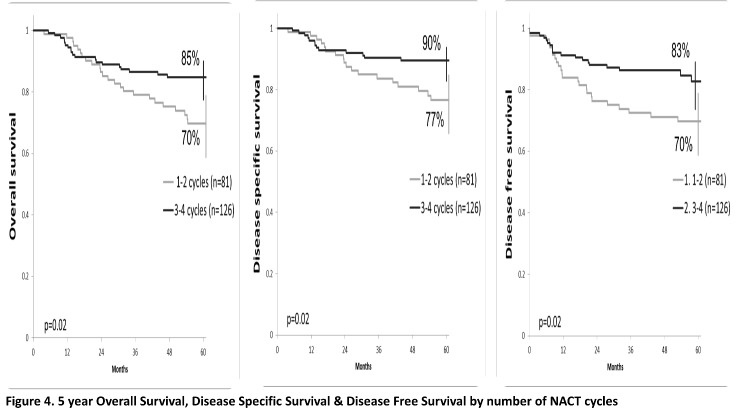
5-year Overall Survival, Disease-Specific Survival & Disease-Free Survival by the number of NACT cycles

At a median follow-up of 58.3 months (ranging from 41.9 to 78.6), 163 (78.7%) patients were alive, 154 patients with CR (74.4%), 49 (23.7%) patients developed recurrence; local recurrence in 17 patients and distant in 32 patients (10 patients had both local and distant failure). The 5-year OS, DSS and disease-free survival (DFS) were 79%, 84% and 78%, respectively. Patients who underwent radical surgery had a statistically significant better OS (87% vs. 72%, p=0.01), SS (90% vs. 80%, p=0.04) and DFS (86% vs. 72%, p=0.02) compared to those who performed exclusive RCT (**Figure 1**). The overall survival for stages IIB and IIIA was 84%, 61% for stage IIIB (p<0.01), SS rates were 90%, 86% and 72% for stage IIB, IIIA and IIIB, respectively (p=0.05). DFS rates were 88% for stage IIB, 76% for IIIA and 69 % for IIIB (p=0.04) (**Figure 2**). According to the OR to NACT, a significantly improved 5-year survival rate compared to the SD patients (OS p<0.01, SS p=0.02, DFS p=0.01) (**Figure 3**). Patients who received 3-4 cycles of NACT had better OS, SS and DFS rates. (p=0.02) (**Figure 4**).

**Fig. 5:**
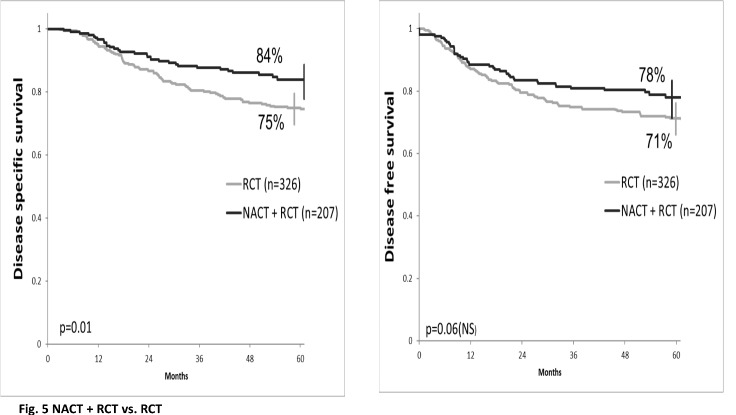
Disease-Specific Survival & Disease-Free Survival comparison between NACT + RCT and RCT

We compared the results of NACT and RCT with the results of another institutional study published in 2012 [8] with radiochemotherapy. The NACT associated with RCT improves DSS and DFS compared to the standard treatment (84% vs. 75%, p=0.01 and 78% vs. 71%, p=0.06, respectively) (**Figure 5**).

## Discussion

The tumor size is an important prognostic factor in cervical carcinoma. Large tumors tend to be less radiosensitive as they have relatively large hypoxic tumor cell population and are thus likely to respond poorly to radiotherapy. To overcome these shortcomings, NACT followed by concurrent chemoradiation is being adopted for a bulky locally advanced disease. Several studies showed that the neoadjuvant chemotherapy is effective in reducing the tumor size, expediting the elimination of micrometastasis, improving operability and surgical downstaging. Even though there are many published trials with NACT before surgery, and recent data from several trials of NACT before RCT became available, no NACT regimen emerged [**9**][**10**][**11**]. All NACT regimes are platinum-based. OR rates of up to 95% were obtained with the administration of paclitaxel combined with either cisplatin or carboplatin. Park et al. reported a 39.5% CR, 11.6% pCR and 51.2% PR after the administration of three courses of paclitaxel and cisplatin in patients with stage IB2-IIB cervical cancer [**8**]. In a prospective phase II study including topotecan and cisplatin as NACT in locally advanced cervical cancer (stage IB2-IIIB), Manci et al. [**12**] showed a 15.8% pCR and 73.7% pPR after the administration of three consecutive cycles of NACT. In a phase II study of dose-dense neoadjuvant chemotherapy (NACT) with paclitaxel and carboplatin before radical chemoradiation (RCT), McCormack et al.showed a complete/partial response rate of 70% at the end of NACT, and 85% after completing RCT [**13**].

In our study, we obtained an overall objective response to NACT of56.5%; 59.9% OR for the PC regimen, and 36.7% OR for the TC regimen, respectively. PC was found to be superior to TC in terms of OR with a 23% difference, (p=0.02). The superiority of the PC regimen was also revealed by the correlation between pCR and the administration of NACT: a 62.5% pCR after the administration of PC and a 53.8% pCR after the administration of TC, but without statistical significance (p=0.56). For the patients who underwent exclusive RCT, the CR rate was 22.9% for PC regimen and 0 for TC. The CR to exclusive RCT in our study was obtained in 19.7% of the 112 patients that underwent RCT. The pCR observed in the 85 patients that underwent surgery was 61.2% (52 patients).

Between 1990 and 2000, a number of non-randomized and randomized studies explored the use of neoadjuvant chemotherapy (NACT) prior to radiation in patients with locally advanced cervix cancer (IIB–IVA). A variety of chemotherapy regimens have been evaluated for use in NACT. Cisplatin-based combinations were used in 8 randomized studies. An improvement in progression-free and overall survival compared to radiation alone has failed to be shown in these studies [**14**][**15**][**16**][**17**][**18**][**19**][**20**].In two studies, the outcome was inferior in the NACT group [**21**][**22**]. In their study, McCormack et al. achieved an overall and progression-free survival rate at 3 years of 67% (95% CI: 51–79) and 68% (95% CI: 51–79) [**12**].In a retrospective study of NACT followed by RCT in locally advanced cervical cancer (stage IB-IVA), Harsh et al. [**23**] showed that 5-year OS and DFS rates were 79.7% and 76.6% for stage II-B, 67.6 and 59.5% for stage III-A, 48.4 and 41.9% for stage III-B.

In our study, the 5 year OS, DSS and DFS were 78%, 84% and 79%, respectively. The OS for stages IIB and IIIA was 84%, and for stage IIIB 61%. DSS rates were 90% for stage IIB, 86% for stage IIIA and 72%for stage IIIB, while DFS rates were 88%, 76% and 69% for IIB, IIIA and IIIB, respectively. Our randomized study, published in 2009 [**24**], confirmed the superiority of cisplatin-based RCT compared to RT alone with 5-year survival rates of 74% vs. 64% (p <0.05). The association of surgery with RCT achieved an 86% OS rate. Another institutional randomized study, published in 2012 [**8**], comparing two radiochemotherapy (RCT) regimens revealed a 75% DSS and 71% DFS at 5 years. The 5-year DSS was 83% in stage IIB, 69% in stage IIIA, and 63% in stage IIIB, while DFS was 83%, 64%, and 53%, respectively. The addition of NACT to standard treatment seems to bring a benefit in DSS (84% vs. 75%, p=0.01) and DFS (78% vs. 71%, p=0.06). A randomized trial is necessary to confirm these data.

The role of surgery in advanced stages is rarely analyzed in the literature. In our study, superior results were obtained by RCT associated with surgery with regard to survival and local control, but it must be mentioned that the patients in stage IIB were optionally operated but in stage III, only the patients with a favorable local response underwent surgery. The evaluation of a surgery combination with RCT is necessary in a randomized study.

## Conclusion

Neoadjuvant chemotherapy followed by concurrent radiochemotherapy seems to bring better results regarding DSS and DFS compared to RCT, but a larger number and long-term evaluation trials are necessary in order to confirm these data.

**Disclosures**

The authors declare that there is no conflict of interest.
